# Additional Antiepileptic Mechanisms of Levetiracetam in Lithium-Pilocarpine Treated Rats

**DOI:** 10.1371/journal.pone.0076735

**Published:** 2013-10-03

**Authors:** Muhammad Y. Al-Shorbagy, Bahia M. El Sayeh, Dalaal M. Abdallah

**Affiliations:** Department of Pharmacology and Toxicology, Faculty of Pharmacy, Cairo University, Cairo, Egypt; Kaohsiung Chang Gung Memorial Hospital, Taiwan

## Abstract

Several studies have addressed the antiepileptic mechanisms of levetiracetam (LEV); however, its effect on catecholamines and the inflammatory mediators that play a role in epilepsy remain elusive. In the current work, lithium (Li) pretreated animals were administered LEV (500 mg/kg i.p) 30 min before the induction of convulsions by pilocarpine (PIL). Li-PIL-induced seizures were accompanied by increased levels of hippocampal prostaglandin (PG) E_2_, myeloperoxidase (MPO), tumor necrosis factor-α, and interleukin-10. Moreover, it markedly elevated hippocampal lipid peroxides and nitric oxide levels, while it inhibited the glutathione content. Li-PIL also reduced hippocampal noradrenaline, as well as dopamine contents. Pretreatment with LEV protected against Li-PIL-induced seizures, where it suppressed the severity and delayed the onset of seizures in Li-PIL treated rats. Moreover, LEV reduced PGE_2_ and MPO, yet it did not affect the level of both cytokines in the hippocampus. LEV also normalized hippocampal noradrenaline, dopamine, glutathione, lipid peroxides, and nitric oxide contents. In conclusion, alongside its antioxidant property, LEV anticonvulsive effect involves catecholamines restoration, as well as inhibition of PGE_2_, MPO, and nitric oxide.

## Introduction

Levetiracetam (LEV) is a unique broad-spectrum second-generation antiepileptic drug (AED), which is used clinically as monotherapy or an add-on drug [1,2]. This AED is employed for partial onset/refractory seizures with or without secondary generalization, juvenile myoclonic seizures, and primary idiopathic generalized tonic-clonic seizures [2]. Experimentally, LEV antiepileptic effect has been documented in amygdala kindling, as well as genetic and spontaneous recurrent seizure models [3–5]. In status epilepticus (SE) model, LEV also delayed the onset of convulsive activity and reduced neuronal injury in pilocarpine (PIL) model, yet it did not affect the ictal discharge [6]. LEV antiepileptic effect resides in inhibition of excessive synchronized activity between neurons [2,7]. Unlike other AEDs, and by virtue of its novel structure, LEV suppresses calcium (Ca^2+^) mobilization from endoplasmic reticulum via binding to neuronal synaptic vesicle protein (SV) 2A [8,9]. Moreover, LEV indirectly impedes neuronal release of glutamate [10], consequently halting excitotoxicity and cellular injury [11]. Such effects are achieved by reducing high-voltage-activated Ca^2+^ current by blocking N- and P/Q-type Ca^2+^ channels [10,12] and suppressing rectifier potassium current [13] resulting in neuronal hyperpolarization. Directly, to maintain a dominant inhibitory environment, LEV affects GABA-receptor mediated currents and opposes the action of negative modulators of GABA and glycine receptors [14]. Furthermore, in kindled animals, LEV downregulates the overexpressed brain-derived neurotrophic factor and neuropeptide Y and increases neuropeptide Y1- and 5-like receptors [5], hence, preventing central modulation of seizure activity.

Inflammation has been implicated in epileptogenesis [15], where elevated levels of cytokines are associated with increased seizure susceptibility [16,17] imposing its role the initiation of neuronal excitability [18]. At the level of the blood brain barrier (BBB), tumor necrosis factor (TNF)-α contributes to the inflammatory response by increasing the expression of selectins and adhesion molecules to recruit leukocytes from the periphery, promoting their adhesion and entry into the CNS parenchyma [19,20]. Moreover, during the acute phase of SE, proinflammatory cytokines transcriptionally activate cyclooxygenase (COX)-2, and hence increases prostaglandin (PG) formation [21]. Free radicals produced during PGE_2_ synthesis, dopamine catabolism, and from activated infiltrated neutrophils might, in part, disrupt glutamate transporters increasing glutamate levels and thus increase seizure susceptibility [22–25]. Besides, catecholamines intimately control epileptic seizures [26,27], where noradrenaline was reported to sustain the activation of locus caeruleus neurons, which limit the spread of seizure during ictal initiation and/or propagation [27,28]. Furthermore, animals lacking a functional noradrenergic system are generally more susceptible to seizures, emphasizing the anticonvulsant role for endogenous noradrenaline [29]. In addition, increased synaptic dopamine concentration inhibits Ca^2+^ influx and suppresses seizure via the activation of D_2_ receptors to reduce N-type Ca^2+^ channels current [26,30–32]. However, to date, the mechanistic pathways of LEV to prevent early seizure onset of SE has not been fully delineated, therefore, the present study aimed to investigate the potential role of some inflammatory mediators, free radical-induced injury, as well as catecholamines on the LEV anticonvulsant effect using the lithium (Li)-PIL-induced seizure model in rats.

## Material and Methods

### 1: Ethics Statement

The study was performed in accordance to the ethical procedures and policies approved by Animal Care and Use Committee of Faculty of Pharmacy, Cairo University and complies with the Guide for the Care and Use of Laboratory Animals [33].

### 2: Animals

Adult male Wistar rats (180±20 g) obtained from El Nile Pharmaceutical Company (Cairo, Egypt) were used. Rats were allowed one week acclimatization period at the animal facility of the Faculty of Pharmacy, Cairo University (Cairo, Egypt). Animals were housed in groups at constant temperature (23±2°C), humidity (60±10%), and a light/dark (12/12 h) cycle with lights on at 5:00 am. They were allowed free access to food and water throughout the experimental period. Seizure induction was done from 9 am to 12 pm to minimize circadian influences on seizure susceptibility.

### 3: Experimental design, seizure assessment, and tissue sampling

Rats were allocated into 3 groups of 12 animals each. In the first group, animals were given saline (i.p.) to serve as control group. In the other two groups rats received lithium chloride (LiCl_3_; 3 mEq/kg i.p.; Sigma-Aldrich, MO, USA) 20 h before the induction of convulsions by PIL (single i.p.; 150 mg/kg; Sigma-Aldrich, MO, USA) [34] or were administered LEV (500 mg/kg i.p.; Sigma-Aldrich, MO, USA) [35] 30 min before PIL to serve as Li-PIL non treated or LEV treated groups, respectively. Immediately after PIL injection, rats were placed singly in Plexiglas cages and were observed for 30 min. During the observation period, convulsive attacks were measured on Racine scale [36]: 0-behavioral arrest (motionless), hair raising, excitement, and rapid breathing; 1-mouth movements (lips and tongue), vibrissae movements, and salivation; 2-head and eye clonus; 3-forelimb clonus; “wet dog shakes”; 4-clonic rearing; 5-clonic rearing with loss of postural control and uncontrollable jumping. LEV treated animals that did not seize within the observation period were given a latency of 30 min. The median of the seizure stages for Li-PIL and LEV treated groups were calculated and the latency onset of the first seizure (stage 3-5), as well as the incidence of convulsing animals were recorded. Afterwards, each of the three groups was further divided into two subsets (n=6 rats). Animals were euthanized under deep ether anesthesia, brains were segregated and the two hippocampi were dissected. Hippocampi in the first subset were homogenized in ice cold saline for the estimation of cytokines, catecholamines, and redox biomarkers. In the second subset, one hippocampus was homogenized in hexadecyltrimethylammonium bromide (1%) in potassium phosphate buffer (100 mM, pH 6) for the determination of myeloperoxidase (MPO) activity, while the other was homogenized in 0.1 M phosphate buffer (pH 7.4), containing 1mM EDTA and 0.1 µM indomethacin for the PGE_2_ measurement. Homogenates were frozen at −80°C for subsequent analysis.

### 4: Determination of TNF-α, IL-10, PGE_2_, and catecholamines

Hippocampal TNF-α, interleukin (IL)-10, and PGE_2_ (R & D Systems, MN, USA), as well as noradrenaline and dopamine (Labor Diagnostika Nord GmbH & Co. KG; Nordhorn, Germany) were assessed using rat ELISA kits.

### 5: Determination of MPO activity

The enzyme activity was conducted according to the method of Bradley et al. [37], where hippocampal homogenates were subjected to 3 freeze/thaw cycles, 10 sec sonication, and 15 min 10,000×g 4°C centrifugation. o-Dianisidine hydrochloride (0.167%)/hydrogen peroxide (0.0005%) in phosphate buffer (50 mM, pH 6) were added to the supernatants. The absorbance rate was recorded at 460 nm.

### 6: Determination of glutathione

Glutathione was assessed using Ellman’s reagent as described by Beutler et al. [38]. Hippocampal homogenates were deproteinated with 5-sulfuosalicylic acid (10%) for 30 min at 4°C, centrifuged at 1000×g for 15 min at 4°C. 5,5′-Dithiobis-2-nitrobenzoic acid (1 mM) was added to the supernatant and the optical density was determined at 412 nm.

### 7: Determination of thiobarbituric acid reactive substances (TBARS)

The thiobarbituric acid reaction of Fee and Teitelbaum [39] was adopted for the estimation of lipid peroxides level, using malondialdehyde as a standard. To hippocampal homogenates, a 1:3 mixture of thiobarbituric acid (0.8%) and trichloroacetic acid (20%) were added and heated for 20 min at 100°C. After cooling, samples were centrifuged at 1000×g for 5 min and the absorbance of the TBARS in the supernatant was read at 535 nm.

### 8: Determination of nitric oxide

Nitric oxide content was quantified indirectly as nitrite/nitrate concentration using Griess reaction dependent method [40]. Hippocampal homogenates were deproteinated with zinc sulphate (30%) for 48 h at 4°C and centrifuged at 12,000×g for 15 min at 4°C. To the supernatant vanadium trichloride (0.8%) in 1 M HCl was added for the reduction of nitrate into nitrite, followed by the rapid addition of Griess reagent [N-(1-naphthyl)ethylenediamine dihydrochloride (0.1%) and sulfanilamide (2%) in HCl (5%)]. The mixture was incubated for 30 min at 37°C, allowed to cool, and the absorbance at 540 nm was determined.

### 9: Statistical analysis

For nonparametric data, values are median of 12 rats and statistical comparisons were analyzed using Kruskal–Wallis test (nonparametric one-way analysis of variance; ANOVA) followed by Dunn’s multiple comparisons test. For stage 3-5 seizure incidence, the Fisher’s exact probability test was used. Parametric data are expressed as mean (n=6) ± S.E.M. and statistical comparisons were carried out using one-way ANOVA followed by Student–Newman–Keuls multiple comparisons test. The minimal level of significance was identified at *P*<0.05.

## Results

Table 1 shows that all Li-PIL treated rats exhibited seizure activity of Grade 3-5 within 5 min after PIL injection. This was manifested as bilateral forelimb myoclonus without (Grade 3) or with (Grade 4) rearing, or with loss of postural control (Grade 5). LEV successfully delayed the seizure latency to reach 27 min as compared to Li-PIL treated rats and profoundly attenuated seizure severity by 83% (Table 1).

**Table 1:** Effect of Levetiracetam (LEV, 500 mg/kg i.p.) on lithium-pilocarpine (Li-PIL) -induced seizures in rats.

**Groups**	**Median seizure stage (minimum – maximum)**	**Stage 3 seizure latency (min)**	**Stage 3 to 5 seizure incidence (%)**
**Li-PIL**	5 (3-5)^*^	5.167±0.207^a^	100^*^
**LEV500**	1 (0-3)^@^	26.958±2.069^b^	16.7^*^,^@^

In the seizure stage, values are median of 12 rats; statistical comparisons were carried out using Kruskal–Wallis test (nonparametric ANOVA) followed by Dunn’s multiple comparisons test. In the parametric analysis (stage 3-5 seizure latency), values are means of 12 animals ± S.E.M.; statistical comparisons were carried out using one-way ANOVA followed by Student–Newman–Keuls multiple comparisons test. Stage 3-5 seizure incidence (12 animals) was compared using Fisher’s exact probability test as compared to control (^*^) and Li-PIL (^@^ groups, *P*<0.05.

**Figure 1:**
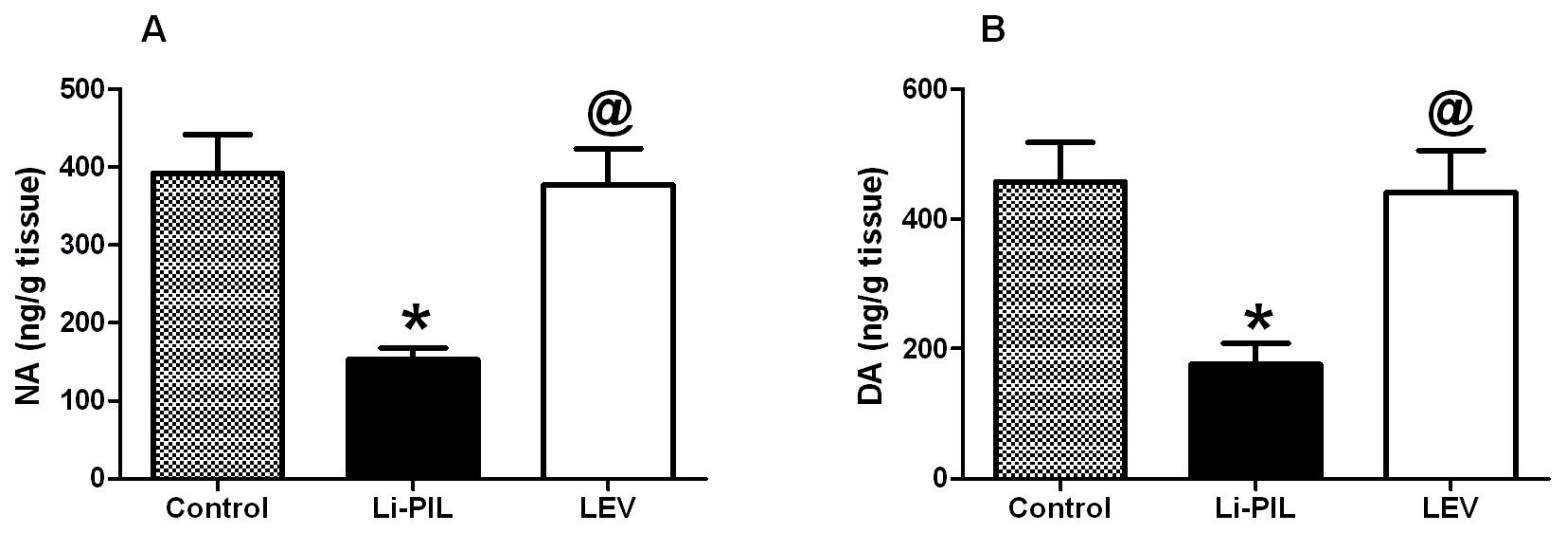
Effect of levetiracetam (LEV, 500 mg/kg i.p.) on hippocampal noradrenaline (NA; A) and dopamine (DA; B) in lithium-pilocarpine (Li-PIL)-induced convulsion in rats. Values are means ± S.E.M. of 6 rats as compared to control (*) and Li-PIL (^@^) groups. Comparisons were carried out using one-way ANOVA followed by Student–Newman–Keuls Multiple Comparisons Test, *P*<0.05.

**Figure 2:**
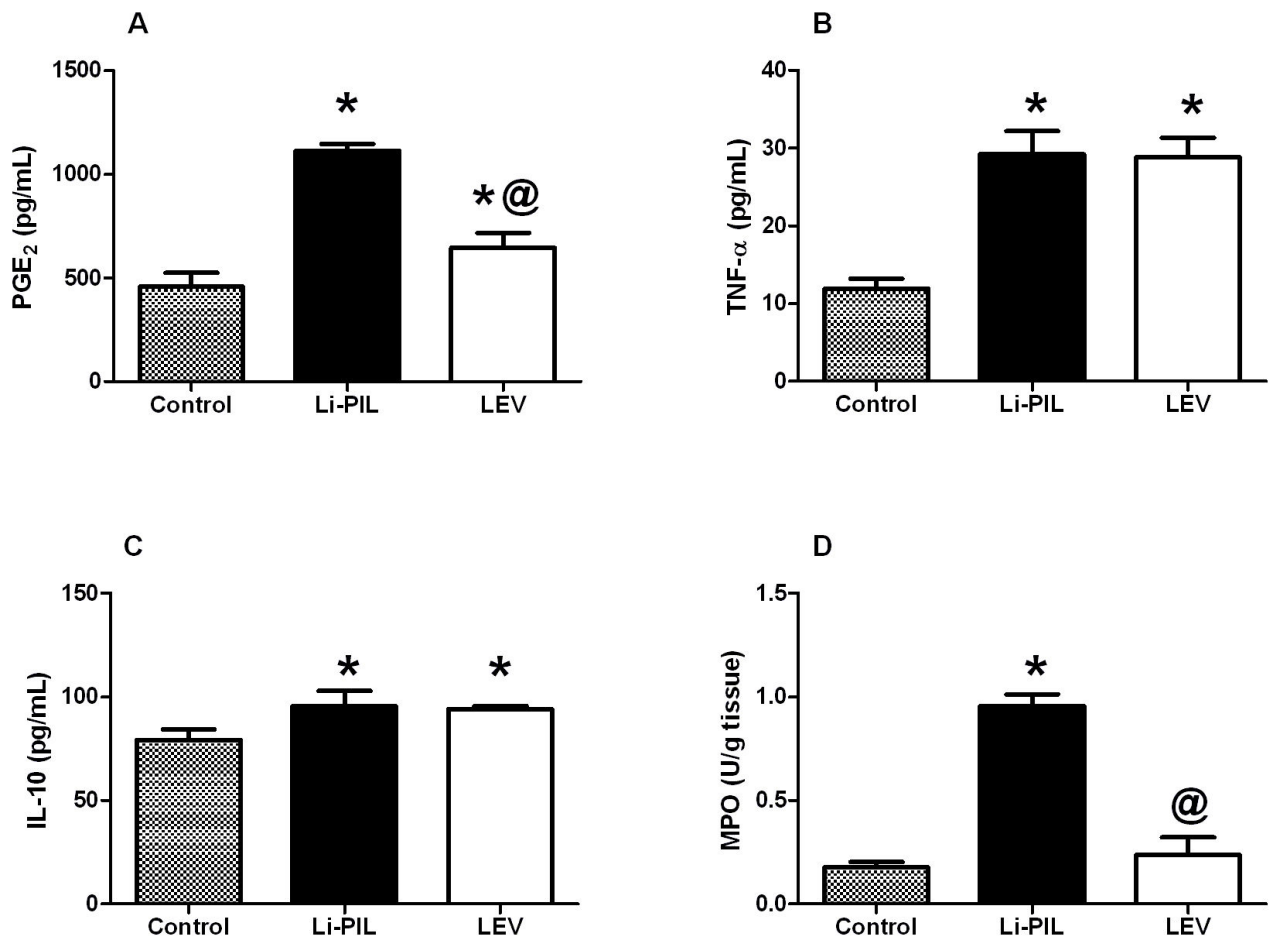
Effect of levetiracetam (LEV, 500 mg/kg i.p.) on hippocampal prostaglandin (PG)E_2_ (A), tumor necrosis factor (TNF)-α (B), interleukin (IL)-10 activity (C), and myeloperoxidase (MPO) (D) in lithium-pilocarpine (Li-PIL)-induced convulsion in rats. Values are means ± S.E.M. of 6 rats. As compared to control (*) and Li-PIL (^@^) groups. Comparisons were carried out using one-way ANOVA followed by Student–Newman–Keuls Multiple Comparisons Test, *P*<0.05.

**Figure 3:**
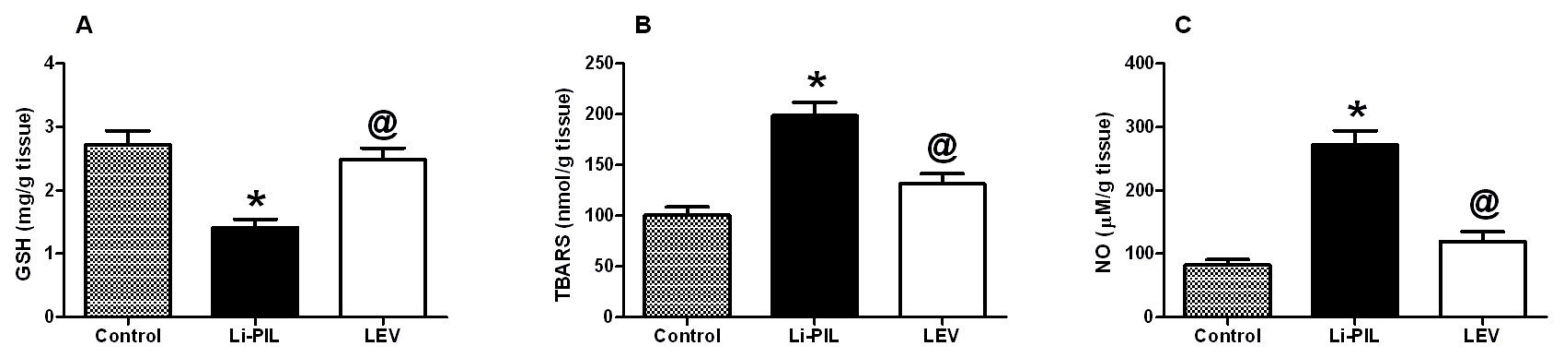
Effect of levetiracetam (LEV, 500 mg/kg i.p.) on hippocampal glutathione (GSH; A), thiobarbituric acid reactive substances (TBARS; B), and nitric oxide (NO; C) in lithium-pilocarpine (Li-PIL)-induced convulsion in rats. Values are means ± S.E.M. of 6 rats. As compared to control (*) and Li-PIL (^@^) groups. Comparisons were carried out using one-way ANOVA followed by Student–Newman–Keuls Multiple Comparisons Test, *P*<0.05.

Regarding the effect on catecholamines, Li-PIL decreased both noradrenaline and dopamine in the hippocampus by about 60% (Figure 1A and 1B), as compared to the control animals, effects that were opposed by LEV pretreatment. Moreover, the hippocampal PGE_2_ level was increased by 2.4 folds upon Li-PIL treatment, as compared to control rats. This effect was halted by LEV pre-administration (57%), as compared to Li-PIL treated animals (Figure 2A). Li-PIL model also elevated TNF-α by 145% (Figure 2B), IL-10 by 21% (Figure 2C), and MPO by 400% (Figure 2D) above the normal levels; however, pretreatment with LEV exerted no effect on the cytokines level, but normalized that of MPO. Furthermore, Li-PIL disrupted antioxidant/pro-oxidant balance in the hippocampus by decreasing glutathione (48%; Figure 3A) and elevating lipid peroxides (TBARS; 97%; Figure 3B), as well as nitric oxide (130%; Figure 3C). These effects were completely prevented by the pre-administration of LEV. 

## Discussion

LEV Antiepileptic Efficacy Is Documented in Clinical as Well as in Experimental Settings Including Li-PIL SE Seizure Model [1,2,4–6]. The Current Investigation Demonstrates That LEV Prevents Li-PIL-Induced Seizures and Reduces the Ictal Incidence in about 80% of Animals. The Anticonvulsant Potential of LEV Involves the Preservation of Hippocampal Noradrenaline and Dopamine Levels, besides Anti-Inflammatory Activity via Inhibition of Neutrophil Recruitment (MPO) and PGE_2_ Synthesis in Li-PIL Treated Rats. In Addition, LEV Antioxidant Property, Detected Previously [27,28] and in the Present Work, Can Add Also to Its Anticonvulsant Effect

During convulsion, neuronal damage is induced by free radicals that are derived from different sources including activated infiltrated neutrophils [22], peroxynitrite formed from nitric oxide interaction with superoxide anion [41], as well as during PGE_2_ synthesis [23] and dopamine catabolism [24]. This investigation emphasized the previous findings, where we report elevated levels of MPO, signifying neutrophil infiltration, nitric oxide, and PGE_2_, along with a decrease in the dopamine level in the hippocampus of Li-PIL treated rats. In seized animals, increased free radicals formation is evidenced by the depletion of glutathione simultaneously with an increase of lipid peroxides, as reported in the current study. These effects are in line with previous studies reporting an imbalance in the redox status in association with seizure activity in PIL model [42,43].

In the present work, LEV antioxidant potential is evidenced by preserving hippocampal glutathione, as well as reducing nitric oxide and lipid peroxide levels. These findings coincide with those of Oliveira et al. [42] and Marini et al. [44], who showed that LEV protects against PIL and kainic acid-induced seizures via its antioxidant potential. Although LEV has no direct antioxidant effect, yet it enhances the effect of the endogenous antioxidants, viz., ascorbate and α-tocopherol [45], events that could account for the decreased lipid peroxidation and enriched glutathione pool in the hippocampus after LEV treatment as shown in the current study. In addition, the LEV-induced restoration of glutathione can be attributed to its up-regulating action on cystine/glutamate exchanger and hence increasing cysteine, the glutathione precursor [45]. Moreover, LEV-mediated inhibition of neutrophil recruitment offers an additional explanation for the reduced free radicals formation. Marchi et al. [46] reported a disruption in BBB permeability after Li-PIL administration that is sufficient to elicit seizures, an effect that aids in the neutrophil infiltration. The reduction in MPO by LEV could be linked to its ability to maintain the BBB integrity [47], where LEV preserves the morphological and functional properties of the BBB together with the reduction of pinocytotic activity during epileptic seizures [48].

Noradrenaline plays a crucial role in suppression of seizure activity, since its depletion increases seizure susceptibility and enhances epileptogenesis [49,50]. Previous studies revealed that agents that normalize noradrenaline exert antiepileptic efficacies [51,52], results that confirm the present findings. The enhancement of noradrenaline by the anticonvulsant could be attributed to increased synthesis and/or reduction in its metabolism as previously elucidated in a PIL seizure model [52], a fact that may entail the effect of LEV on dopamine. Notably, β_2_ adrenergic receptor activation increases hippocampal dopamine level that stimulates D_2_ receptor to prevent epileptogenesis [53,54]. Meanwhile, the activation of α_1A_ adrenergic receptor increases hippocampal GABA to inhibit limbic seizures [54]. The latter event aggregates additively to halt excitotoxicity [55] giving a further insight to the anticonvulsant effect of LEV.

Suppression of excitotoxicity by LEV is reflected in the current investigation by the reduction in hippocampal PGE_2_ and nitric oxide. These excitotoxicity hallmarks were altered following Li-PIL administration that corroborate with previous findings [42,56,57]. Both free radicals and TNF-α transcriptionally up-regulate COX-2 to form PGE_2_ during which reactive oxygen species are produced [21,23,58]. LEV-mediated inhibition of PGE_2_ may be linked to inhibition of glutamate release [10], LEV-induced antioxidant activity and/or noradrenaline enhancement, rather than the suppression of TNF-α, which was not affected by LEV in this study. The AED neither exerts its anticonvulsant effect via modulation of TNF-α nor its counter partner IL-10 that is adaptively elevated during epileptogenesis induced by Li-PIL as previously reported [15,59].

Taken all together, besides abrogating oxidative/nitroactive stress, LEV anticonvulsant effect is additionally attributed to normalization of noradrenaline and dopamine, inhibition of PGE_2_ and MPO.
